# The rising burden of chronic conditions among urban poor: a three-year follow-up survey in Bengaluru, India

**DOI:** 10.1186/s12913-015-0999-5

**Published:** 2015-08-15

**Authors:** Mrunalini J Gowda, Upendra Bhojani, Narayanan Devadasan, Thriveni S Beerenahally

**Affiliations:** Institute of Public Health, 250, 2nd C Main, Girinagar 1st Phase, Bengaluru, 560085 India; Department of Public Health, Ghent University, De Pintelaan 185 4K3 9000, Ghent, Belgium

## Abstract

**Background:**

Chronic conditions are on rise globally and in India. Prevailing intra-urban inequities in access to healthcare services compounds the problems faced by urban poor. This paper reports the trends in self-reported prevalence of chronic conditions and health-seeking pattern among residents of a poor urban neighborhood in south India.

**Methods:**

A cross sectional survey of 1099 households (5340 individuals) was conducted using a structured questionnaire. The prevalence and health-seeking pattern for chronic conditions in general and for hypertension and diabetes in particular were assessed and compared with a survey conducted in the same community three years ago. The predictors of prevalence and health-seeking pattern were analyzed through a multivariable logistic regression analysis.

**Results:**

The overall self-reported prevalence of chronic conditions was 12 %, with hypertension (7 %) and diabetes (5.8 %) being the common conditions. The self-reported prevalence of chronic conditions increased by 3.8 percentage point over a period of three years (OR: 1.5). Older people, women and people living below the poverty line had greater odds of having chronic conditions across the two studies compared. Majority of patients (89.3 %) sought care from private health facilities indicating a decrease by 8.7 percentage points in use of government health facility compared to the earlier study (OR: 0.5). Patients seeking care from super specialty hospitals and those living below the poverty line were more likely to seek care from government health facilities.

**Conclusion:**

There is need to strengthen health services with a preferential focus on government services to assure affordable care for chronic conditions to urban poor.

## Background

Globally 36 million people died in 2008 due to non-communicable diseases, one of the major contributors to chronic conditions [1]. Nearly 80 % of these deaths occurred in low- and middle-income countries [1]. The prevalence of chronic conditions is on the rise globally as well as in India. In 2014, 60 % of all the deaths in India were due to non-communicable diseases and the burden is estimated to increase over the time [2–4]. Urbanization is linked with greater risk for and burden of major chronic conditions. While India is urbanizing at a rapid pace, there is a huge intra-urban inequity with urban poor having poorer access to basic amenities and poorer health indicators compared to the affluent urbanites [5].

The recent studies reveal high prevalence of chronic conditions among urban poor in India [6–9]. While it remains contentious whether, in a strict epidemiological sense, the poor in India suffer greater burden from chronic conditions compared to rich, there seems to be consensus that they form a highly vulnerable group that needs urgent attention in terms of care and control of chronic conditions [10–20]. In this context, it is crucial to monitor trends in prevalence and health-seeking for chronic conditions among urban poor.

As part of the community-based action research project, we had conducted a census in a poor urban neighborhood in Bengaluru (India) in 2009–2010. That study revealed a high burden of chronic conditions among residents [7]. Subsequently, The Urban Health Action Research Project trained three community health assistants from the same community to strengthen the existing health systems and also create awareness about the chronic conditions and its management. We conducted a follow-up survey after three years in the same population. This paper reports findings from the follow-up survey, and compares it with the earlier study to develop trends in prevalence and health-seeking behavior among residents over the time.

## Methods

### Study setting

KG Halli is the field site of the Urban Health Action Research Project (UHARP) being implemented by the Institute of Public Health in Kadugondanahalli (KG Halli) since 2009. KG Halli was purposefully selected for the UHARP to study how access to quality healthcare could be improved in a poor urban community with a pluralistic healthcare system. A cross sectional survey was conducted to understand self reported illness and health seeking profile. The residents as well as healthcare providers in KG Halli have identified unaffordable healthcare expenses as one of the major issues in the area [21]. The institutional ethics committee from Institute of Public Health, Bengaluru, India approved this study.

KG Halli is one of the 198 administrative units of Bangalore, a metropolitan capital of Karnataka. KG Halli has a population of over 44,500 individuals in an area of less than a square kilometer. KG Halli has two recognized slums and comprises of people from Karnataka as well as migrants from other Indian states. Majority of the population in the community are daily wageworkers. KG Halli has a mixed healthcare system with two government facilities run by municipal and state government and around 32 private healthcare facilities. Services offered by government facilities are heavily subsidized and, in principle, free for people living below the poverty line. Private facilities that include many single-doctor clinics and four hospitals work on fee-for-service basis.

### Sample size

Considering the 8.6 % of overall prevalence of self-reported chronic condition as found in the earlier study in KG Halli [7], 95 % confidence interval and 1 % of precision, we estimated the minimum sample size needed for our survey to be 3286 individuals. We added another 50 % of this number in order to cover for non-response. A few factors made us to account for high non-response rate.

The population of KG Halli comprises largely of migrants who often keep shifting their residence. In the course of our project activities and the earlier survey, we would find many households empty or closed for long time. Also the community in the area is weary of participating in surveys – as they often are approached by various agencies dealing with marketing of commercial products and/or as part of welfare projects/schemes. Majority of adults in the community are daily-wage workers who are often not at home during the day. There are many nuclear families where all the adults might be at work and so it’s likely that such households will not have an adult respondent at home when data collectors approached the houses. Considering the average household size of 4.7, we aimed to survey a minimum of 1047 households in KG Halli.

### Data collection and measurements

As part of the UHARP project, a baseline census was conducted in 2009–2010 to understand the socio- demographics and health related aspects of the community. We conducted a follow-up survey in 2012–2013 in KG Halli to monitor changes in socio-demography, prevalence of self-reported illness, health-seeking and healthcare expenditure. The baseline survey revealed a high prevalence of self reported chronic conditions, especially that of diabetes and hypertension. These patients were incurring high out of pocket expenses from these conditions [7]. As part of the action research project varied strategies were employed, three community health assistants were identified from the same community and were trained for over a period of one year. The community health assistants started conducting regular house-to-house visits creating awareness on chronic conditions in general and for diabetes and hypertension in particular. They directed them to appropriate healthcare services in the area. Periodic meetings with healthcare providers in the area were conducted to discuss health issues of the population identified form the baseline survey and also provide local solutions for the same. In this paper we selectively analyze these parameters in reference to chronic conditions. Community health workers collected data at household level using a structured questionnaire. They administered a questionnaire to available and willing family member aged 18 years or above. They selected every tenth family in a sequential order, starting from the Vinobhanagar area, a southern end of KG Halli. In case of refusal or unavailability of eligible respondent, the immediate next household replaced the household. The data collectors took informed verbal consent from the participants before administering the questionnaire. The completed questionnaires were verified and revisits to surveyed households were made on the following day for any corrections or missing data. The research team verified 10 % of the questionnaires from the survey. The methods for the survey including the tool for data collection were similar to those used for the baseline census. While we briefly outline methods used for this survey, kindly refer to the earlier publication [7] for detailed data collection method.

Three binary outcome variables were defined for assessing prevalence of chronic conditions. These were the ‘absence’ (coded as ‘0’) or ‘presence’ (coded as ‘1’) of: i) any chronic condition, ii) diabetes and iii) hypertension. We considered a chronic condition to be present when a respondent reported having prescribed or taking medications on a daily basis for at least 30 days preceding the survey. A chronic condition is defined as an illness or impairment that lasts for a long duration. The minimum time period for an illness to be considered chronic varies depending on the source of definition, ranging from three months to one year [7]. The names of chronic conditions were initially recorded using the lay terms reported by respondents and later categorized by the researchers, to the extent possible, into specific conditions. Based on the names of the reported chronic conditions, the presence or absence of diabetes and hypertension were also recorded.

Similarly, three binary outcome variables were defined to assess health seeking for chronic conditions. These were type of health services sought (‘private’ coded as ‘0’, ‘government’ coded as ‘1’) for: i) a chronic condition, ii) diabetes and iii) hypertension. For this study, we coded the outcome variable based on the nature of the health facility where the first consultation occurred. We compared values of these outcome variables with the findings from baseline census conducted three years ago.

Apart from comparison with the earlier study, we examined association of these outcome variables with a set of predictor variables. Predictor variables included sex (‘male’ or ‘female’), age (transformed into three age groups: (0- ≤ 40; > 40- ≤ 60; > 60), per capita income per month (as income quintiles), religion (‘Islam’, ‘Hindu’, and ‘Christian’) and the household poverty status (‘above’ or ‘below’ the poverty line) as established by the type of ration card (a proof of identity which establishes the economic status of a family) possessed by the household. While examining predictors for health seeking, we included an additional predictor in form of the tier of the healthcare services sought. Three tiers of healthcare services were defined based on where the person with a chronic condition was being managed at the time of the survey: i) ‘clinics/health centers’, ii) ‘referral hospitals’ with in-patient facilities and iii) ‘super-specialty hospitals’ attached to medical schools. Though there are overlaps in the provision of services across clinics/health centers, referral hospitals and super-specialty hospitals, they roughly correspond to primary, secondary and tertiary healthcare services, respectively.

### Data analysis

The data were entered using EpiData Entry software 3.1 (The EpiData Association, Odense, Denmark). The data was checked for errors and missing values before being analyzed using STATA 11.2 (StataCorp, Texas, USA).

The prevalence of self-reported chronic conditions is reported as a percentage with 95 % confidence interval. To identify the predictors of self-reported chronic conditions, a multivariable logistic regression model was developed using all aforementioned predictors. The interaction between predictor variables was checked and two-way interaction terms that were significant at p < 0.05 were included in a multivariable logistic regression model. Similar to a backward elimination technique, the predictors that were not significant at p < 0.05 were then dropped sequentially while comparing models for goodness of fit (using a likelihood-ratio test) until no further improvement was possible. A similar process was used to develop the final multivariable models for all other outcome variables. Multi colinearity was assessed by using post-estimation commands. The final models are represented with the adjusted odds ratio (OR), 95 % confidence interval and p values.

## Results

In total, we surveyed 1099 households or 5340 individuals, well over the minimum sample size estimated. We achieved 95 % response rate. The non-response (5 %) was either due to refusal to respond (3 %) or absence of an eligible respondent in the household (2 %) at the time of the visit. Table 1 provides socio-demographic characteristics of the study population.

**Table 1:** Socio-demographic features of sample population

Characteristics		Baseline survey (N = 44,514)	Follow-up (N = 5,340)
Sex: N (%)	Male	22,702 (51.0)	2,760 (51.7)
	Female	21,801 (49.0)	2,580 (48.3
Age group:	< 19 years	17,335 (39.0)	1,993 (36.1)
	20–39 years	17,140 (38.5	1,958 (36.6)
	> 40 years	10,013 (22.5)	1,388 [27]
Per capita income: per month in INR median(interquartile range)	1^st^ quintile (poorest)	1,200 (1000–1285.7)	500 (500–692)
	2^nd^ quintile	1,625 (1500–1750)	916 (714–1100)
	3^rd^ quintile	2,000 (2000–2250)	1,250 (1111–1428)
	4^th^ quintile	2,875 (2531.3–3200)	1,750 (1500–2153
	5^th^ quintile (least poor)	5,000 (4000–6142.9)	3,000 (2222–20,000)
Religion: N (%)	Islam	30,481 (68.7)	3,788 (71)
	Hindu	9,317 (21.0)	1,022 (19.1)
	Christian	4,569 (10.3)	501 (9.3)
Household poverty status: N (%)^a^	Above the poverty line	23,442 (52.7)	2,715 (50.8)
	Below the poverty line	4,783 (10.7)	569 (10.6)

^a^Total does not add up to 100 because several households accounting for 38.6 % of sample population did not possess ration card

In the sample population 12 % individuals reported to be living with one or more chronic conditions. Hypertension (6.2 %) and Diabetes (4 %) were two most commonly reported chronic conditions. The other chronic conditions which were also reported in the community were thyroid (0.7 %), heart problem (0.7 %) and leg pain (0.6 %). Presence of more than one chronic condition (comorbidity) was reported by 4 % in the community.

Compared to the earlier study in the same population, our findings indicate a significant increase in self-reported chronic conditions in KG Halli. There was 3.8 percentage point increase in prevalence of overall chronic conditions with 1.5 times greater odds of reporting chronic conditions among population in 2012–2013 compared to three years ago. The baseline survey revealed that 3 % of patients with self-reported chronic conditions were not on treatment [7]. In the follow-up survey, the treatment gap increased by 0.3 %. Table 2 provides comparison of prevalence rates and health-seeking pattern for overall chronic conditions and for hypertension and diabetes.

**Table 2:** Comparison of prevalence rates and health-seeking behavior in two cross-sectional surveys for chronic diseases, diabetes and hypertension

	Self-reported prevalence rate		Absolute difference in percentage points	Odds ratio with 95 % confidence interval
	2009–2010^a^	2012–2013		
	N = 44514	N = 5340		
Chronic conditions	8.6 %	12 %	3.8	1.5 (1.4, 1.6)
Diabetes	4 %	5.8 %	1.8	1.5 (1.3, 1.7)
Hypertension	6.2 %	7.1 %	0.9	1.2 (1.0, 1.3)
	Health-seeking from government sector			
Chronic conditions	19.4 %	10.7 %	−8.7	0.5 (0.4, 0.7)
Diabetes	14.8 %	7.2 %	−7.6	0.4 (0.3, 0.7)
Hypertension	18.1 %	9.5 %	−8.6	0.5 (0.3, 0.7)

^a^Source: Bhojani et al. [7]

Table 3 depicts the predictors for overall self-reported chronic conditions, diabetes and hypertension. Increase in age was associated with significant increase in odds of reporting any chronic condition including diabetes and hypertension. Women had greater odds of reporting chronic conditions. While this remained true for prevalence of hypertension, sex did not appear to be a significant predictor for diabetes. Association between per-capita income (in form of income quintiles) and prevalence rates of chronic conditions was not statistically significant.

**Table 3:** Predictors of prevalence of self-reported chronic conditions, diabetes and hypertension

Predictor variables^a^	Overall chronic conditions N = 637		Diabetes N = 312		Hypertension N = 379	
	Unadjusted odds ratio (95 % CI)	Adjusted odds ratio (95 % CI)	Unadjusted odds ratio (95 % CI)	Adjusted odds ratio (95 % CI)	Unadjusted odds ratio (95 % CI)	Adjusted odds ratio (95 % CI)
Sex						
Men	-	-				-
Women	1.62	1.79	1.28	1.32	1.91	1.93^b^
	(1.3,1.9)	(1.3, 2.4)	(1.0,1.6)	(1.0,1.9)	(1.5,2.3)	(1.3,2.7)
Age groups (years)						
0 - ≤ 40	-	-		-		-
> 40 - ≤ 60	9.6	21.97	20.56	26.79		21.69^b^
	(4.6,20.0)	(15.8,30.4)	(2.7,153.3)	(15.5,46.2)		(14.1,33.2)
> 60	178.64	49.14	528.42	58.81		48.93^b^
	(88.4,360.6)	(30.8,78.3)	(74.0,3769.1)	(31.3,110.4)		(28.6,83.5)
Monthly per capita income						
First quintile		-		-		
Second quintile	1.29	1.41	1.32	2.03	1.23	1.25
	(0.9,1.7)	(0.8,2.2)	(0.8,2.0)	(1.1,3.7)	(0.8,1.8)	(0.7,2.1)
Third quintile	1.17	0.96	1.10	0.98	1.00	0.86
	(0.8,1.6)	(0.5,1.5)	(0.6,1.7)	(0.5,1.9)	(0.6,1.5)	(0.4,1.5)
Fourth quintile	1.24	1.14	1.11	1.26	1.15	1.09
	(0.9,1.6)	(0.7,1.8)	(0.7,1.7)	(0.6,2.3)	(0.7,1.7)	(0.6,1.8)
Fifth quintile	1.62	1.31	1.71	1.42	1.48	1.22
	(1.2,2.1)	(0.8,2.0)	(1.1,2.6)	(0.7,2.5)	(1.0,2.1)	(0.7,2.0)
Household poverty status						
Above the poverty line	-	-	-	-	-	-
Below the poverty line	0.84(0.63–1.11)	0.99	0.66	0.58	0.86	1.07
		(0.6,1.4)	(0.4,1.0)	(0.3, 1.0)	(0.6,1.2)	(0.6,1.6)
Religion						
Islam	-	-	-	-	-	-
Hinduism	1.12(0.9–1.3)	0.74	1.23(0.9–1.6)	-	0.87(0.6–1.1)	0.53 (0.3,0.8)
		(0.5,1.0)				
Christianity	1.21(0.9–1.5)	1.14	0.99(0.6–1.5)	-	1.02(0.7–1.5)	0.91 (0.5,1.5)
		(0.7,1.8)				

^a^For all the predictor variables, the first category mentioned serves as the referent category. Absence of data against certain predictor variables suggests that those variables were not part of the final model arrived at during multivariable logistic regression for prevalence of respective category of chronic conditions

^b^Predictor variable is significant at p< 0.05

When compared to the earlier study [7] (data not reproduced in this paper), an increase in age, being woman and living below the poverty line emerge as common positive predictors for self-reported overall prevalence of chronic condition. Unlike the earlier study, we did not find significant difference in prevalence of overall chronic conditions across different income quintiles. While comparing self-reported prevalence of diabetes across the two studies, increase in age appeared as the common positive predictor. Though living below the poverty line was associated with lesser odds of reporting diabetes in both the studies, the association in the current study was statistically not significant (p = 0.05). Unlike earlier study, sex did not appear as significant predictor for diabetes. While comparing prevalence of hypertension, increase in age and women appeared as common positive predictors. While living below the poverty line was associated with greater odds of reporting hypertension in both the studies, the association was statistically not significant in the current study.

In our sample population, 89.3 % of people reporting chronic conditions sought care from private sector. Similarly 92.8 % with diabetes and 90.5 % with hypertension sought care from private sector. While the earlier study also indicated that the majority of people with chronic conditions sought care from private sector, the current study shows that the proportion of people seeking care from private sector actually increased over the time (See Table 4). The odds of seeking care from government sector reduced by half compared to the earlier study. Close to half of the people with chronic conditions sought care from clinics/health centers (46.6 % with chronic conditions, 48.2 % with diabetes and 51.8 % with hypertension), followed by hospitals (33.9 % with chronic conditions, 36.9 % with diabetes and 30.8 % with hypertension) and super specialty hospitals (19.5 % with chronic conditions, 14.5 % with diabetes and 17.4 % with hypertension).

**Table 4:** Predictors for seeking healthcare from government facilities

Predictor variables	Overall chronic conditions N = 637	Diabetes N = 312	Hypertension N = 379	
	Adjusted odds ratio (95 % CI)	Adjusted odds ratio (95 % CI)	Adjusted odds ratio (95 % CI)	
Sex^a^	-	-	-	-
Age^a^	-	-	-	-
Monthly per capita income				
First quintile	-	-	-	-
	-			
Second quintile	0.52	0.05	0.58	
	(0.1, 1.8)	(0.0, 0.6)	(1.1,2.8)	
Third quintile	1.39	0.36	1.34	
	(0.4, 4.3)	(0.0, 2.8)	(0.2, 6.2)	
Fourth quintile	0.53	0.12	0.53	
	(0.1, 1.8)	(0.1, 1.4)	(0.1,2.8)	
Fifth quintile	0.57	0.15	0.75	
	(0.1, 1.8)	(0.0, 1.0)	(0.1, 3.3)	
Household poverty status				
Above the poverty line	-	-	-	
Below the poverty line	2.59	8.28	2.75	
	(0.9, 6.7)	(1.5, 44.2)	(0.8, 8.6)	
Tiers of health services				
Clinics/health center	-	-	-	
Referral hospitals	1.51	4.78	1.74	
	(0.5,4.4)	(0.7, 29.4)	(0.5,5.8)	
Super specialty hospitals	16.60	9.46	8.67	
	(6.3, 43.3)	(1.0, 82.9)	(2.6,28.7)	
	0.00		0.00	

^a^The predictors variables were not significant fit to the model describing the health seeking behavior hence were not included in our analysis

Predictors of health seeking are depicted in Table 4 People seeking care from super specialty hospitals were significantly more likely to go to government facilities compared to private facilities. Also, people living below the poverty line had greater odds of seeking care from government facilities. These two factors were also found to be positive predictors of health seeking from government sectors in the earlier study, which in addition found that people of and above 60 years were more likely to seek care from government facilities compared to younger age groups.

## Discussion

Self reported prevalence and health-seeking behavior for chronic conditions was compared with a similar survey conducted three years ago in the same population. We found that the prevalence rates of overall self-reported chronic conditions and that of diabetes and hypertension increased significantly in the last three years. Similar to the earlier study, the majority of the patients sought care from private sector and in fact we found significant increase in proportions of patients seeking care from private sector over the time.

The already high and rising prevalence of self-reported chronic conditions among poor is of great concern. Studies in India show that many chronic conditions (like, diabetes and hypertension) remains undiagnosed, and hence the actual prevalence of these conditions in population could be much higher [20, 22]. The awareness provided by the community health assistants about chronic conditions, diabetes and hypertension among people and healthcare providers in KG Halli over the three years would have been one of contributing factors to an increase in reporting of prevalence of self-reported chronic conditions. A separate study conducted in KG Halli in 2012–2013 assessing the knowledge and self-management practices of diabetes patients revealed that the awareness about the disease remains low (unpublished data). Other studies indicate that the actual prevalence of these conditions also seems to be on rise among urban poor. Deepa et al. [23] show that over a decade in Chennai (another metropolis in South India) the prevalence of diabetes and hypertension among urban poor not only increased but increased at a greater rate compared to general urban population.

The rising prevalence combined with greater reliance on private health sector implies a huge economic burden for urban poor. Private sector in India largely works on fee-for-service basis. The earlier study from the study area revealed that out-of-pocket payments by people with chronic conditions for outpatient care doubled the poverty ratio within a month. Due to several reasons including inadequacies of government health sectors, there seems to be general preference for private sector among urban poor [7, 24, 25].

The Government of India recently launched the National Urban Health Mission [26] in order to revamp and improve healthcare for urban poor. The mission among its several activities proposes to provide screening and diagnostic services at primary care level for chronic conditions. The mission itself took much longer time to take off and it would be important that various services for chronic conditions including access to medications and training of healthcare personnel to deal with chronic conditions are integrated into the mission and are implemented to strengthen the government healthcare services. However, considering that the majority of urban poor seek care from private sector at present, the mission needs to ensure that private sector delivers rationale quality care while protecting people from impoverishment due to healthcare payments. While the mission acknowledges the high utilization of private sector, it does not directly addresses issues related to cost and quality of care in this sector. Strengthened primary care will also ensure that most of the people with chronic conditions are diagnosed and managed at this level avoiding burden on referral care facilities while making care for chronic conditions closer, affordable and hopefully people-centered.

Our study has some limitations. We assessed prevalence of self-reported chronic conditions. While self-report of morbidity seems to be somewhat reliable and useful indicator, the prevalence rates would differ when assessed through self-report and bio-medical tests [16, 17]. As indicated earlier, the true prevalence of chronic conditions is likely to be greater than what we report in this study. In terms of health-seeking behavior, we asked people which health facility they went to for seeking care. Based on ownership we classified these facilities into government and private. Health seeking is a complex phenomenon and people often move from government to private sector and vice versa. People often seek care from both the sectors for a single episode of care. For example, a person might visit a government center and see a doctor there. He/she then might visit private laboratory or pharmacy to get diagnostic tests or medications respectively if that is not available at that facility. What we capture in our study is the facility where the primary consultation with doctor happened. Finally, we compare the findings from a census of the entire individual in KG Halli (n = 44154) with that from a survey of a sample population (n = 5340). While we surveyed adequate individuals in order to assess prevalence of chronic conditions in the same population (See Methods), readers shall exercise caution while reading the comparisons.

## Conclusions

There is a high prevalence of self-reported chronic conditions among residents of a poor urban neighborhood in Bengaluru city. The majority of people with chronic conditions seek care from private sector. Both the prevalence of self-reported chronic conditions and preference for private sector increased over the last three years. Many predictors of self-reported chronic conditions and the health-seeking pattern remain same over the time. There is need to pay urgent attention on improving chronic conditions care for urban poor with a preferential focus on strengthening the government primary care services.
